# Sub-Surface Analysis of Grinding Burns with Barkhausen Noise Measurements

**DOI:** 10.3390/ma16010159

**Published:** 2022-12-24

**Authors:** Aki Sorsa, Mika Ruusunen, Suvi Santa-aho, Minnamari Vippola

**Affiliations:** 1 Environmental and Chemical Engineering, University of Oulu, P.O. Box 4300, FI-90014 Oulu, Finland; 2 Materials Science and Environmental Engineering, Tampere University, P.O. Box 589, FI-33014 Tampere, Finland

**Keywords:** Barkhausen noise, residual stress, grinding, grinding burns, sub-surface stress, X-ray diffraction, data analysis

## Abstract

Barkhausen noise (BN) measurements are commonly used for surface characterisation. However, often there is also a need to verify the sub-surface region because detrimental tensile stresses may be present after different manufacturing steps. Especially in a grinding burn, the surface stress may be compressive, but it changes quickly into tensile stress below the surface. The aim of this study was to find out whether regular surface-sensitive BN measurement is also sensitive to the stresses below the surface caused by grinding burns. More specifically, the aim was to study the relationship between BN features and sub-surface stresses and to identify a model that estimates sub-surface stresses. Real samples were collected from an actual process. The samples were cylindrical samples manufactured from commercial alloyed AISI/SAE L6 steel that was through-hardened prior to grinding. Barkhausen noise measurements were carried out for 42 grinding burn locations followed by X-ray diffraction-based residual stress surface measurements and residual stress depth profiles. Depth information was obtained through step-by-step electrolytic removal of thin layers. The stress profiles were pre-processed through interpolation and averaged stress was calculated as a function of depth below the surface. Correlation analysis was carried out to evaluate the relationships between BN features and stress at different depths and among BN features. The main outcome of the analysis was that BN measurement is dominated by the sub-surface tensile stresses rather than the compressive stress at the surface. It was also noticed that BN features form two groups, corresponding to average Barkhausen activity and magnetising field strength leading to maximum Barkhausen activity. Models for stress at different steps were identified systematically. The performance of the models for sub-surface stresses was reasonable with *R*^2^ values of around 0.85 and root mean squared error (RMSE) values of around 95 MPa. Based on the results, it is concluded that BN measurement provides information about sub-surface stresses and that stress can be evaluated through straightforward modelling, allowing fast detection of grinding burns.

## 1. Introduction

Grinding is a common method for creating the final surface finish for hardened components and is usually used in demanding components, such as various gears and other transmission components. As is well known, the grinding process is highly dependent on grinding wheel performance, selection and conditioning [1] as well as on the correct grinding parameters for the specific case. During grinding, the variation in different grinding parameters can introduce different temperatures within the grinding zone and, if non-optimal grinding parameters are used, the heat input produced might be too high and cause grinding burns. For example, a locally increased material removal rate may lead to the risk of grinding burn [2]. As Malkin and Guo [3] have stated, most grinding damage is thermal in origin. A temperature rise may cause either local microstructural phase changes or mechanical property variations that might even result in the relaxation of stresses [3].

Typical grinding burns are temper burns occurring at lower grinding contact zone temperatures and re-hardening burns that occur at higher temperatures, producing untempered martensite during cooling [4]. Temper burns appear when the temperature exceeds the original tempering temperature, typically 450 °C [5] and re-hardening burns appear when the temperature exceeds the austenitisation temperature (above *Ac*_3_) and phase change occurs. Temper burns do not necessarily contain visual burn marks or temper colours, as stated in [5]. However, their appearance in the micrograph might look like increased dark areas near the surface where the diffusion of carbon has taken place. Usually, softening is noticed from microhardness profile measurements and the damage may extend to a depth of up to 100 µm. Rowe [5] states that softening is a problem with hardened steels, but not typically with low-carbon steels. Regarding re-hardening burns, a hard and brittle martensitic white layer can be seen at the surface from micrographs and above a softened layer where the temperature rise was lower.

Both types of grinding burns mentioned above will compromise the material properties and may lead to failure during the use of the ground pieces. Koster et al. [6] point out that the presence of untempered martensite and overtempered martensite in hardened steel is equally detrimental to the surface integrity of hardened steel because both will cause a decrease in fatigue strength. Additionally, the residual stress (RS) state at the surface and the sub-surface will be affected by the grinding burn formation. In a typical milder grinding burn, the surface stress may be compressive (but lower compared to the original) but it changes quickly into tensile stress below the surface. However, if the grinding parameters are chosen progressively, an even tensile stress can be formed at the surface [2]. Therefore, different grinding parameters have a great effect on the outcome of the grinding, such as the material removal rate (*Q_w_*), which is the volume rate per unit of time at which the material is removed from a workpiece during grinding, and material removal (*V_w_*), which is the volume of workpiece material removed during the grinding time. 

It is also evident that surface residual stress value evaluation alone might not be enough to determine the grinding burn. As described in [7], RS depth profiles were made from ground gear flanks that had nearly the same compressive residual stresses at the surface. However, the stress values diverged just below the surface and showed the largest variation in tensile residual stresses 0.02 mm below the surface. At that depth, the residual stresses had a good correlation with the corresponding surface Barkhausen noise (BN) values (the highest BN corresponded to the highest tensile residual stress peak below the surface). In [2], small gears manufactured from 20MnCr5 were studied with RS depth profiles. The grinding burns were due to rising material removal rates (*Q_w_*), which generated the higher thermal loads that were found with production BN measurements. In the RS depth studies of these gear samples, it was noticed that the tensile residual stresses occurred only at depths greater than 20 µm and the residual stresses in the surface remained compressive.

Sackmann et al. [8] studied RS depth profiles from ground samples. They observed the effect of the material removal rate on the RS depth profiles. It was noticed that a removal rate of 6 mm^3^/mms produced a recognised tensile residual stress peak around 10 µm below the surface and that higher material removal rates led to higher tensile residual stresses below the surface and shifted the maximum tensile stress to deeper below the surface. The highest *Q_w_* of 24 mm^3^/mms led to a 750 MPa stress peak at a depth of 50 µm. The highest *Q_w_* also produced a re-hardening layer and strong tempering underneath, which was verified from the micrograph. Light tempering was noticed with a *Q_w_* of 12 mm^3^/mms.

One commonly utilised quality control method for detecting the surface changes caused by grinding is the magnetic non-destructive Barkhausen noise (BN) method. Nowadays, Barkhausen noise measurement has an established position in the industry for grinding burn detection [2]. However, there is also often a need in the industrial processes to verify the sub-surface region because detrimental tensile stresses may be present as a result of different manufacturing steps. Commonly, BN-based systems are utilised in the grinding burn inspection carried out after the process. The surface is scanned for BN measurement and areas with higher BN readings indicating possible grinding burns are detected. Often only a single BN feature such as the root mean square (RMS) value is monitored. 

A better approach for grinding burn detection would be to use multiple BN features [8]. Such an approach could even allow the identification or severity evaluation of the detected grinding burns. In addition, modelling has been studied for improving grinding burn detection. For example, Heinzel et al. [9] combined thermal modelling and experimental grinding studies to determine the critical residual stress state at the surface via BN measurements. Some very recent publications have utilised BN-based systems for in-process monitoring purposes inside the grinding machine [10,11]. Such an approach allows modification of the grinding parameters during processing and thus would produce more consistent surface quality. 

As mentioned above, identification of sub-surface stresses is important for detecting grinding burns. BN measurement is an intriguing method for stress detection because its features have been shown to correlate with stress and material properties in general. The root mean square (RMS) value is sensitive to changes in the stress state of the studied material and shows a good correlation, as, for example, in [12,13], with a certain range of stresses. Beyond this range, the residual stress vs. RMS correlation has also been shown to have saturating behaviour when the tensile or compressive stress exceeds a certain limit [14]. Generally, the RMS value increases with increasing tensile stress (or decreasing compressive stress). In [14], the reverse relationship was observed when the aforementioned saturation point was exceeded. In [15], a linear relationship is reported between the applied stress and the reciprocal of the BN peak amplitude (i.e., peak height). In addition to the challenges above, the hardness of the material may determine the stress dependence: for harder material, the trend for RMS and stress was observed to be linear, whereas for softer material the trend was found to be exponential in the studies of Santa-aho et al. [16]. In addition, in some cases the trend between the measured RMS value from ground components and the surface residual stress correlation has been rather low, for example, 0.46 [17]. 

This study considers grinding burn detection after processing and uses real samples from an actual process. The main research question in this study is whether surface BN measurement is sensitive to sub-surface residual stresses. This question is answered with a correlation analysis that shows the correlations of multiple BN features with the RS as a function of depth below the surface. A correlation analysis between BN features aims to provide further insight into significant BN features in terms of grinding burn detection. The final aim of this study is to build a model that estimates the sub-surface RS with the features from a surface BN measurement. The model gives further evidence for the main research question stated above and also shows the potential of using multiple BN features rather than just one. Even though the model identified here shows that the prediction of sub-surface RS is possible, it is used here more as a data analysis tool. Thus, model accuracy is not the main issue and only a simple linear regression model is identified. 

## 2. Materials and Methods

### 2.1. Samples

Real ground samples were collected from an actual grinding process. The samples were cylindrical samples manufactured from commercial low-alloyed steel, AISI/SAE L6, that was through-hardened prior to grinding. After grinding, production line BN measurements were taken with a Rollscan 200 (manufactured by Stresstech Oy, Finland) to identify test pieces with local grinding burns with ViewScan software. Commercial BN sensor fitting to a cylindrical shape was utilised. In total, 42 grinding burn locations were identified by the industrial quality control system. Grinding burns in these locations were verified by characterisation with BN MicroScan measurements, surface residual stress measurements and residual stress depth profiles.

### 2.2. Barkhausen Noise Measurements

**Figure 1:**
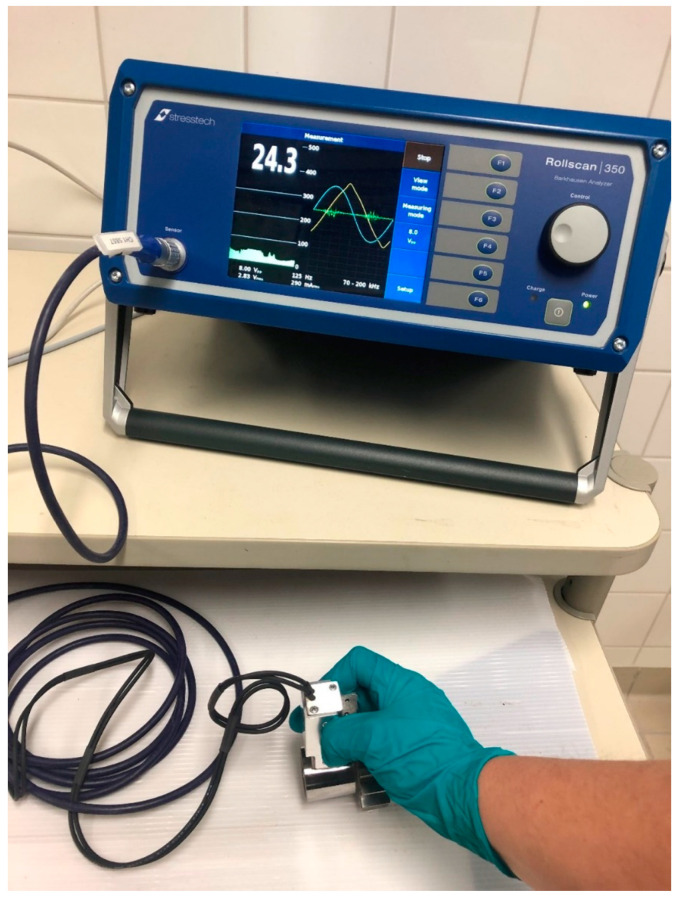
Barkhausen noise measurement set up.

The multiparameter Barkhausen noise measurements were carried out with a Rollscan 350 BN measurement device using MicroScan software (Stresstech Oy, Finland). A commercial S5857 outer diameter (OD) sensor (Stresstech Oy, Finland) was utilised. The measuring direction for BN was the tangential direction of the cylindrical samples. The magnetising voltage was 8 V_PP_ (voltage peak-to-peak) and the magnetising frequency was 125 Hz. An analysing frequency range of 70–200 kHz was utilised and MicroScan software was used to record the BN features. The measurement set up is shown in Figure 1. 

### 2.3. Residual Stress Measurements

The residual stress measurements were performed for the ferrite phase using the X-ray diffraction (XRD) technique with a portable XStress 3000 X-ray residual stress diffractometer (manufactured by Stresstech Oy, Finland). In addition to the residual stresses, the full-width-half-maximum (FWHM) of the diffraction peak values was also examined. The measurements were performed using the modified chi-squared method [18] in the tangential and longitudinal directions of the cylindrical samples. The principle of the XRD measurements is that the interplanar lattice spacing of the ferrite [211] plane can be calculated from the reflections from the Bragg’s diffraction angle of 156° using Bragg’s law. The inclined measurements were made at four different tilt angles for both tilt directions. The stress can be indirectly determined from the change in the lattice parameters determined by the different tilt angles when the X-ray elastic constants, i.e., elastic modulus and Poisson’s ratio, are known. The measurements with the XStress 3000 stress analyser were conducted with a collimator of 3 mm, operated using 30 kV voltage, 6.7 mA current and CrKa radiation. CrKa radiation was selected as it gives a measuring depth of 5–6 µm. This is somewhat less than the information depth of BN measurements [19]. 

**Figure 2:**
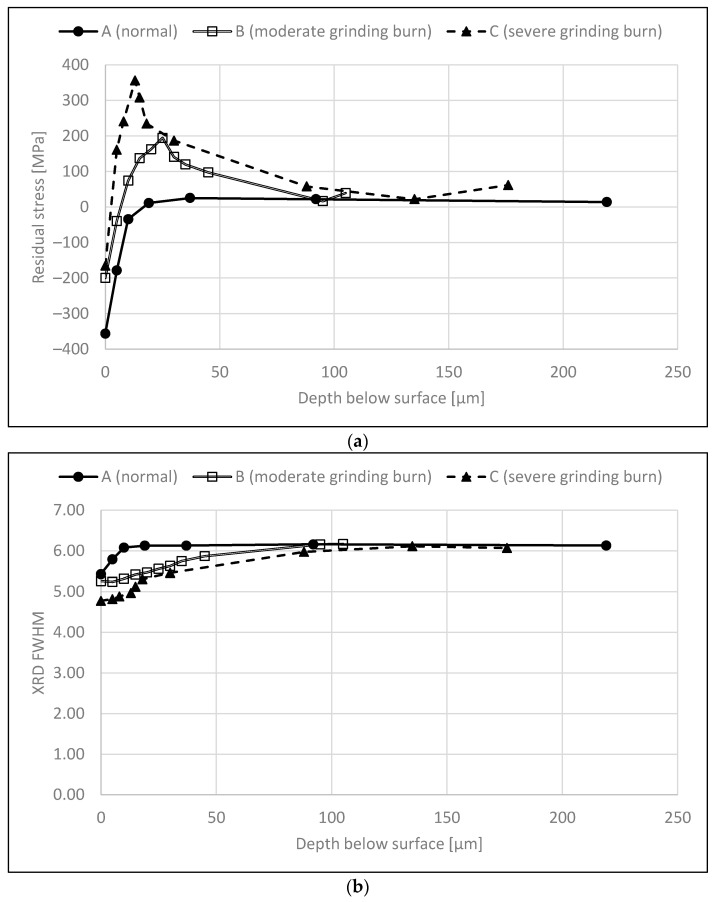
Examples of (**a**) residual stress profiles and (**b**) XRD FWHM profiles. The measurements were carried out in the tangential direction. A refers to a normal profile (surface RMS 60), B refers to a moderate grinding burn (surface RMS 73) and C refers to a severe grinding burn (surface RMS 74).

Residual stress depth information was acquired by the step-by-step electrolytic removal of thin layers with Movipol-3 electrolytic polishing equipment (Struers, Ballerup, Denmark) with commercial Struers A2 perchloric acid solution in a small area with a diameter of 5 mm. The depth of the removed layer was verified using a Mitutoyo dial indicator. Usually, residual stress depth profiles were made deep below the surface where the stress level had been levelled out as a constant value. Figure 2a presents examples of a normal RS depth profile (A), a moderate grinding burn (B) with a 300 MPa tensile stress peak below the surface and a more severe grinding burn (C) with a 600 MPa tensile stress peak below the surface. Similar results were obtained in [2,7,8,20], where almost similar high surface compressive residual stress values were obtained in samples having tensile residual stress peaks beneath the surface and where BN was sensitive to the sub-surface tensile stresses. Figure 2b presents example profiles for the XRD FWHM values. 

The XRD FWHM value, i.e., the broadening of the diffraction peak, is known to correspond to the microstrains and microstresses that are related to the microstructural state of the studied component and thus can also be related to hardness [21]. Besides this information on crystals (nature and symmetry) and dislocation density/plastic deformation [22,23], the XRD FWHM can be associated with changes in stresses in some cases, as studied by Vashista and Paul [22] with ground samples. However, the relation between the XRD FWHM and RS was found to be polynomial, linear or constant depending on the grinding details utilised [22]. In this paper, it is assumed that the XRD FWHM is related mainly to hardness, even though it is acknowledged that it is affected by other factors as well. The assumption is also supported by the analyses given in [7,20]. Figure 2b shows examples of XRD FWHM profiles for normal grinding (A), a moderate grinding burn (B) and a severe grinding burn (C).

### 2.4. Data Pre-Processing

**Figure 3:**
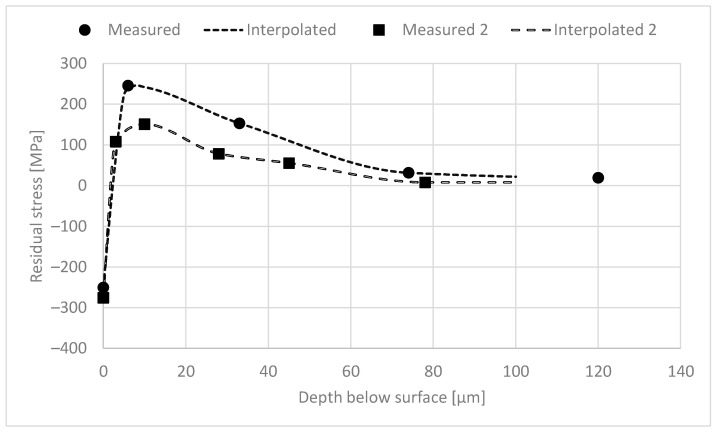
Two examples of RS depth profiles.

The dataset obtained contained the surface BN measurements and RS and XRD FWHM depth profiles of 42 samples. The BN measurement device used computes certain BN features automatically. These features are the RMS value, peak height, position and width, coercivity, remanence, integral area, spectrum area and pulse count. These BN features were used as such, but the depth profiles needed pre-processing. The main issue was that the profiles did not have uniform measurement depths, as shown in Figure 2. Thus, the first pre-processing step was to interpolate the depth profiles to uniform depths. The depth profiles were interpolated between 0 and 100 µm with an increment of 2.5 µm. The main interest of this study lies in the surface and sub-surface region (0–50 µm). Figure 3 shows two examples of the profiles obtained. In the first example, the measurements were carried out for 120 µm while in the second example the last depth measured was only 78 µm. Thus, in the second example extrapolation was needed. All the interpolated profiles were carefully checked; one profile was ignored because extrapolation gave an unreasonable profile.

The second pre-processing step was to produce the data for the analyses. The aim was to process the interpolated profiles so that the datasets held the information desired. Thus, the interpolated RS and XRD FWHM profiles were averaged as a function of depth. The averaging was carried out for 10 µm profile sections. There is no physical explanation for this selection but 10 µm sections gave a dataset of suitable size and were assumed to include the depth dependency of BN features. The surface RS and XRD FWHM values were also included in the datasets. Thus, the datasets obtained contained values for the surface and 0–10, 10–20, … and 90–100 µm sections. The measurements were carried out in longitudinal and tangential directions and thus two datasets with 11 RS values and two datasets with 11 XRD FWHM values were obtained. The analyses were carried out for all the datasets, but the results are presented only for the datasets with measurements in the tangential direction. This is because the correlations between the longitudinal and tangential measurements were high (*R*^2^ > 0.92) in this case and thus the results were similar in both directions. It should, however, be noted that this might not be the case for individual samples. 

### 2.5. Data Analysis

The data analysis applied was three-fold. Firstly, the correlation coefficients between the surface BN features and averaged RS and XRD FWHM values were studied to gain knowledge on how the correlations change as a function of depth. Thus, it is possible to evaluate whether grinding burns can be detected with surface BN measurements. Secondly, the correlation coefficients were calculated between BN features. Combined with the results from the first analysis, significant BN features can be identified concerning grinding burn detection. As a third step, a regression analysis was carried out where a simple linear regression model was fitted between BN features and averaged RS and FWHM values. The models identified are given by:

(1) $${\hat{\sigma }}_{i}=b_{0}+\sum_{j=1}^{P}b_{j}x_{i,j}$$

Above, ${\hat{\sigma }}_{i}$ is the estimated RS, i refers to a data point, $b_{0}$ is the bias term, $b_{j}$ are the regression coefficients, $x_{j}$ are the BN features included in the model and *P* is the number of BN features in the model. A linear model in (1) was identified sequentially following these steps:

1. A full model with all the variables was identified.
2. F statistics together with *p*-values were calculated for all model terms.
3. The term with the biggest *p*-value was removed from the model if it was not statistically significant. The α risk for statistical significance was set to 0.05.
4. A model without the removed variables was identified and then returned to step 2.
5. Steps 2–5 were repeated as long as there were statistically insignificant terms in the model.

The performance of the fitted models was evaluated with the *R*^2^ and root mean squared error (RMSE) values. The RMSE value is given by:

(2) $$RMSE=\sqrt{\frac{1}{N}\sum_{i=1}^{N}{\left(\sigma_{i}-{\hat{\sigma }}_{i}\right)}^{2}}$$

Above, *N* is the number of data points. Additionally, a plot of estimated vs. measured RS values was evaluated.

## 3. Results and Discussion

The results are presented here for the XRD measurements carried out in the tangential direction. For clarity, some BN features are excluded from some parts of the analysis. For example, peak height, remanence and spectrum area are not included in the analysis in the following two sections because their behaviour is very similar to that of the RMS value and integral area. The same was also observed in [20,24]. The correlation of pulse count was found to be always low and thus it is excluded as well. 

### 3.1. Analysis of RS as a Function of Depth

**Figure 4:**
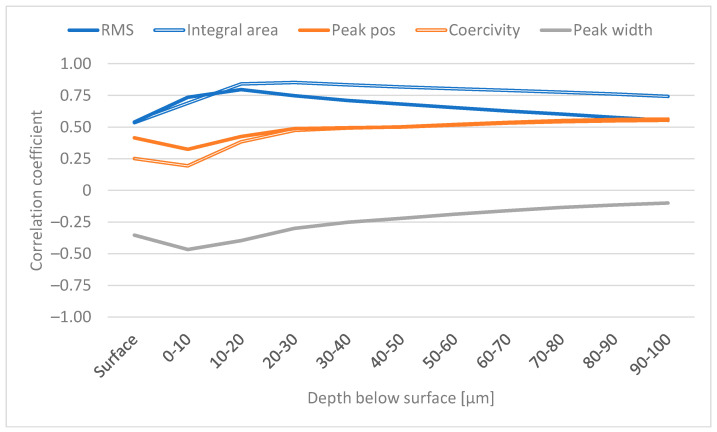
Correlation coefficient between selected BN features and RS (tangential).

Correlation coefficients were calculated between the averaged RS and the BN features. Figure 4 shows these coefficients for selected features. The figure shows that the correlation between all features and RS at the surface is rather low (0.54 at maximum for the RMS value). Generally, the correlations increase as a function of depth. The RMS value and integral area reach their maxima at 10–20 µm and 20–30 µm below the surface, respectively, followed by a subtle decrease. This is to be expected because the samples with grinding burns have compressive stresses at the surface but high tensile stresses in the sub-surface region, as shown in Figure 2a; in addition, it is well known that higher tensile stress (or lower compressive stress) is associated with higher RMS values (for example, [23]). Similar results were also reported in [7]. It is assumed that the sub-surface region with tensile stress dominates the sample and makes a greater contribution to the BN signal compared with the surface. Results indicating the same phenomenon are presented in [7,20] where increased grinding burn damage led to higher tensile stresses in the sub-surface region and increased RMS values. However, it is noted in [8] and shown in the results in [20] that the relationship between tensile stress and the increase in RMS value is not linear; instead, a smaller increase in the RMS value is observed with more severe damage. The decrease in the correlation shown in Figure 4 has two possible explanations. Firstly, the BN signal attenuates the deeper it penetrates [25] and, secondly, the residual stresses approach 0 at increased depths, as shown in Figure 2. Thus, there is less variation in the RS values and the correlations decrease.

The correlation for peak position and coercivity shown in Figure 4 also increases in the sub-surface region, followed by a subtle increase after 30 µm. However, the correlations in general are lower than the correlations with the above-mentioned features. This is probably because peak position and coercivity are often observed to correlate more with hardness and thus the XRD FWHM (for example, [26,27,28]). 

The correlation between peak width and RS reaches its maximum at 0–10 µm and then approaches zero. This behaviour is probably explained by the attenuation of the BN signal as mentioned above. It should be noted that the correlation between peak width and RS is negative.

### 3.2. Analysis of XRD FWHM as a Function of Depth

**Figure 5:**
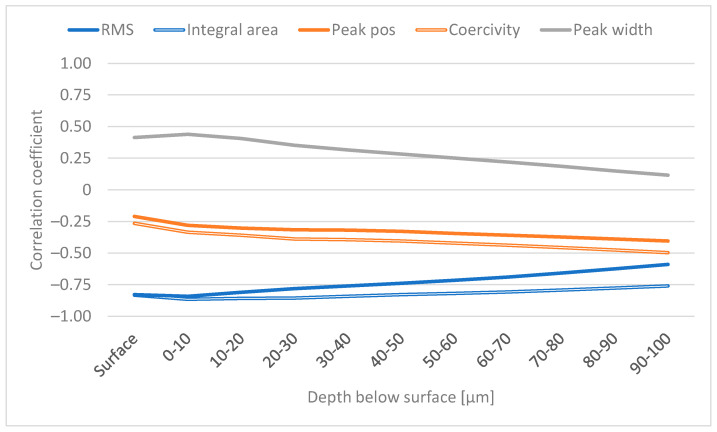
Correlation coefficient between selected BN features and XRD FWHM (tangential).

As shown in Figure 2b, the XRD FWHM profile is gentler than the RS profile shown in Figure 2a. This can also be seen in Figure 5 where the correlations between the averaged XRD FWHM values and the selected BN features are presented. The RMS value and integral area have a strong negative correlation with the XRD FWHM. This is to be expected because hardness (and thus the XRD FWHM) and BN activity are known to have an indirect relationship (for example, [16,26,29]) meaning that higher hardness leads to a decreased BN signal. It is assumed that the softer surface makes a greater contribution to the XRD FWHM value than the layers with increased hardness, which explains the higher correlations near the surface. 

The correlations between the XRD FWHM values and peak position and coercivity are somewhat surprising. Firstly, higher correlations were expected because these features are often related to hardness changes (for example, [26,27,28]) and, secondly, the correlations were expected to be at their highest at or near the surface. Both of these observations can be explained by the samples used. As shown in Figure 2, the samples show greater changes in the RS profiles than in the XRD FWHM profiles especially in the surface and sub-surface regions. Thus, it is to be expected that the data, especially from the surface and sub-surface regions, are dominated by the changes in RS. Thus, correlations for features corresponding to hardness are low for surface and sub-surface regions. 

The correlation of peak width and XRD FWHM behaves similarly to that in Figure 4 but now has a positive correlation. Again, the BN signal attenuation is the probable cause.

### 3.3. Analysis of Significant BN Features

**Figure 6:**
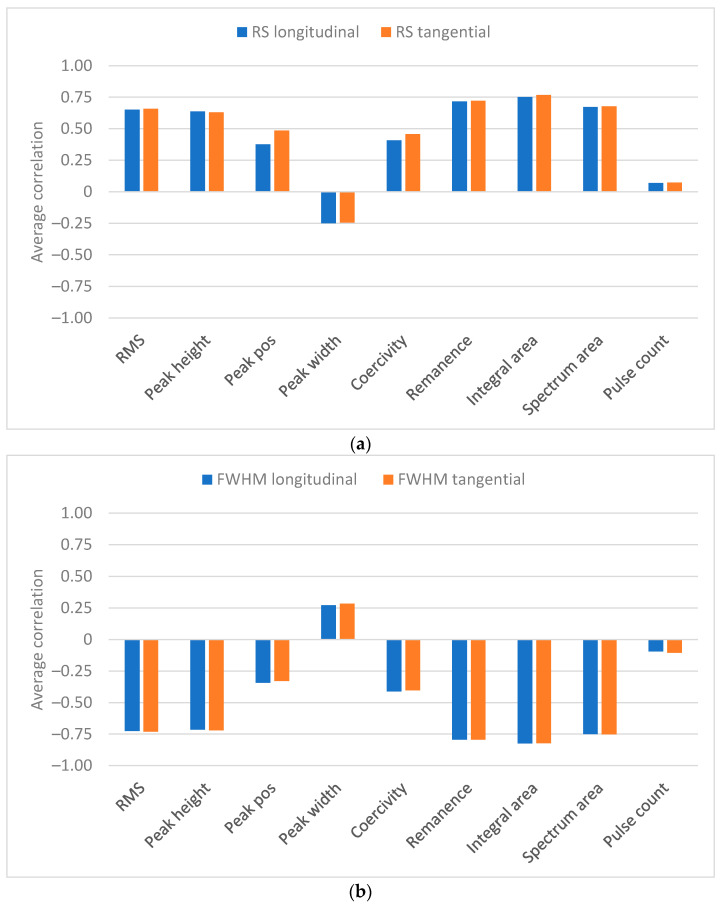
(**a**) Average absolute correlation between BN features and RS. (**b**) Average absolute correlation between BN features and XRD FWHM.

There are a lot of studies in the literature evaluating the significant BN features for estimating RS and hardness. In this study, the direct results in the form of averaged correlation coefficients are presented in Figure 6. The figure shows that the integral area has the highest absolute correlation with RS and XRD FWHM. Remanence has the second highest absolute correlation while the RMS value, peak height and spectrum area are third with almost equal correlations. For all the features mentioned above, the correlation is positive for RS and negative for XRD FWHM. Furthermore, all five features mentioned above have rather high correlations (>0.85) with each other, which has been observed earlier in [20,24]. 

The above-mentioned features excluding remanence indicate average Barkhausen activity. In the literature, mainly the RMS value and peak height have been studied whereas remanence, integral area and spectrum area are not often considered. It has been shown in many studies (for example, [23]) that they have a positive correlation with stress and negative correlation with hardness (for example, [26]). Thus, the results obtained here are well aligned with the literature. The novel information provided in Figure 6 is the strong correlation of the integral area. It is not clear why the correlation is the highest for the integral area. One possible explanation is that, by integrating the BN signal, an approximation of the hysteresis loop is obtained. Thus, it is possible that integral area combines the positive properties of the features associated with the average Barkhausen activity and hysteresis loop parameters (remanence and coercivity). It is also possible that this observation only holds for this dataset. 

Peak position and coercivity have been reported to be closely related [12]. Their correlation in this study was observed to be 0.81, which gives an indication of their close relationship. This is also verified by Figure 4 and Figure 5. It has often been observed that peak position and coercivity correlate with hardness (for example, [26,27,28]) so that higher hardness leads to increased peak position and coercivity values [27]. On the other hand, increased compressive stress has been noticed to increase peak position and coercivity values [12,26,30]. In this study, hardness increases as a function of depth and thus increased peak position and coercivity are to be expected. However, the stress profile shows increasing tensile stress in the sub-surface region and thus lower peak position and coercivity values can be expected. It is possible that these effects cancel each other out and thus the overall correlation of peak position and coercivity is low. 

Generally, peak width has been observed to decrease with increased tensile stress [12,30]. Moreover, in [31], peak width was included in the prediction model of RS with a negative regression coefficient. All the above indicates that the correlation between peak width and stress should be negative. The results in this study show that the correlation between peak width and RS is negative but rather low on average. However, as indicated in Figure 4, higher negative correlations are obtained for the near-surface region. 

The results obtained for pulse count show that its correlations with RS and XRD FWHM are low. Furthermore, the correlation as a function of depth is not only small but also changes its sign. This clearly indicates that pulse count does not hold any useful information about grinding burns.

### 3.4. Estimation of RS in the Sub-Surface Region

Earlier, it was noticed that BN features and RS and XRD FWHM have correlations with each other. Even though this is interesting, a more important question is whether tensile sub-surface stresses can be estimated and thus provide useful information for detecting grinding burns. As mentioned in Section 2.5, a linear regression model given in (1) was identified between BN features and interpolated and averaged RS values. The estimation models were developed for surface, 0–10, 10–20, 20–30, 30–40 and 40–50 µm residual stresses with the approach explained in Section 2.5. Models were limited to 50 µm because the tensile stresses were formed within this sub-surface region and mainly settled at zero deeper below the surface (Figure 2). The models obtained and their performance are presented in Table 1. 

**Figure 7:**
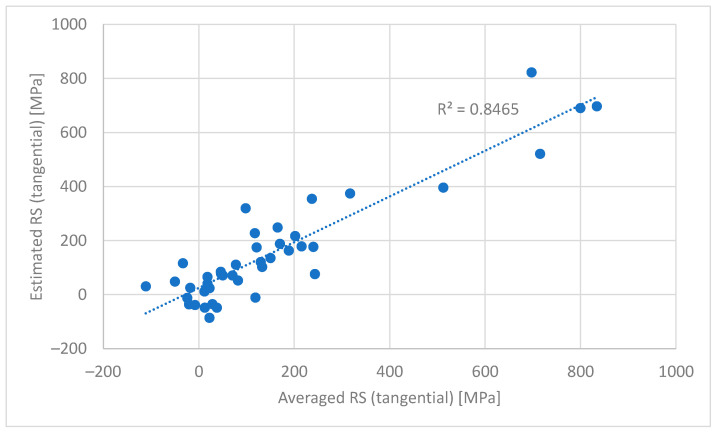
Scatter plot of estimated vs. measured RS for the model with the best performance (20–30 µm). The BN features in this model were the RMS value, peak position, coercivity and integral area.

The first observation in Table 1 is that the estimation model for surface RS is not as good (*R*^2^ = 0.50) as expected based on the earlier analyses. Increasing the depth increases the accuracy of the identified models. The best models are identified for 20–30 µm but the difference from the models for 10–20, 30–40 and 40–50 µm is only marginal. The obtained *R*^2^ values for these model structures are 0.82–0.85. Figure 7 shows the scatter plot of estimated vs. measured RS for the best model. The figure shows that the trend of RS changes is nicely captured by the model. Figure 7 also shows the benefits of the modelling approach presented here compared with the current practice where only elevated BN readings (usually only one BN feature) are observed and components are accepted or rejected based on these. The models refine the information in the measurement data and deeper analysis of the grinding burn can be carried out. 

The second observation regarding Table 1 is that the RMSE values indicating the average modelling error are rather high. For the best models, they are about 95 MPa. One reason for this is that the model estimates only RS but hardness and other material properties also influence BN measurement. The interaction between material properties and BN are complex and thus it is possible that a simple linear regression model is questionable for the modelling task. Thus, more complex model structures could lead to more accurate results. Nevertheless, the uncertainty involved with BN measurement can be high [32] and thus simpler model structures should be preferred. 

The features used in the modelling form two groups. The first includes the RMS value, peak height, remanence, integral area and spectrum area as mentioned in Section 3.2. The second group includes peak position and coercivity. Peak width and pulse count do not correlate significantly with the other features. The analysis of the features included in the models (Table 1) shows that at least one feature from the groups mentioned above is always included. It is no surprise that the first group mentioned above is always represented in the model. The average Barkhausen activity and especially the RMS value are observed to correlate well with stress and many material properties. In [7,20], it is reported that the RMS value increases with increased tensile stresses in the sub-surface region, indicating that it can be used in grinding burn detection. Thus, it is natural that at least one feature from the first group is present. 

Peak position and coercivity are both always included in the models, as shown in Table 1. Considering the correlations presented earlier, it is slightly surprising that these features seem to be significant. However, the correlations evaluate features individually. Then, the analysis of correlations may be misleading for features that are significant together with some other feature. In this case, it seems that peak position and coercivity together are significant and thus they are always in the models together. In [20], it is noted that the RMS value alone cannot be used in grinding burn detection and should be complemented with peak position or coercivity. The analysis of their significance and variation led to the conclusion that coercivity should be preferred [20]. The results presented here indicate that both should be used. However, this observation may hold only for the dataset used and thus further verification is needed. 

It should also be noticed in Table 1 that the number of features included in the model seems to increase with depth. The reason for this is not clear but it is assumed that it is due to the decreasing correlations as a function of depth. Basically, this means that closer to the surface the model includes fewer but stronger features, whereas deeper the models include more but slightly weaker features. It should be borne in mind that the modelling scheme applied does not include model validation and thus the results presented here might be slightly optimistic. However, the number of features in the models is small compared with the number of data points and thus the risk of serious overfitting is low. 

## 4. Conclusions and Further Research

Grinding burn detection with Barkhausen noise measurement was studied in this paper. The samples were collected from a real grinding process and a total of 42 samples with grinding burns were used. Barkhausen noise measurements were carried out from the sample surfaces and residual stress and XRD FWHM profiles were measured. It was noticed from the profiles that tensile stresses appeared within the sub-surface layer (0–50 µm) of the samples. The profile information was complemented through interpolation and profiles were averaged to gain information from certain segments below the sample surface. 

The interpolated and averaged profile information was analysed to gain knowledge about how the relationships between Barkhausen noise features and residual stress and XRD FWHM changed as a function of depth below the surface. It was noticed that the relationships between residual stress and BN features were at their strongest at 20–40 µm below the surface. For XRD FWHM and BN features, the highest correlations were noticed at or near the surface. Average correlation coefficients were calculated to gain knowledge about the most significant BN features and the relationships between the BN features. It was found that BN features formed two groups, where the first group contained the RMS value, peak height, remanence, integral area and spectrum area and the second group included peak position and coercivity. In general, all the features categorised as the first group had a strong correlation with residual stress and XRD FWHM while the correlation of the second group was lower. 

To gain knowledge about the tensile stress in the sub-surface region, simple regression models were identified between residual stress and BN features. Models were identified for residual stress values at a certain depth from the surface. The identified models contained features that are in good agreement with the literature. The estimation models captured the main changes in residual stress (*R*^2^ around 0.85) in the sub-surface region well, even though the average modelling error (RMSE) was observed to be around 95 MPa, which was considered quite high. Even though the model structure applied was simple, valuable information concerning the detection of grinding burns can be obtained with the approach applied in this study especially compared with the current practice where only increased Barkhausen activity is observed.

This study gave some results that need further verification. The next step in the research should be to build classification models which can distinguish between good and faulty components. The dataset applied in this study is not adequate for this task because it contains only samples with grinding burns. Thus, a dataset with both good and faulty samples is required. The classification model could be further refined by classifying not only the faulty samples but also the fault types based on their severity.

## Figures and Tables

**Table 1:** Models obtained for estimating averaged residual stresses.

Depth below Surface (µm)	BN Features	*R* ^2^	RMSE (MPa)
Surface	Peak position, Coercivity, Spectrum area	0.50	139.6
0–10	Peak position, Coercivity, Spectrum area	0.68	120.3
10–20	Peak position, Coercivity, Integral area	0.83	95.7
20–30	RMS value, Peak position, Coercivity, Integral area	0.85	94.2
30–40	Peak height, Peak position, Coercivity, Integral area, Spectrum area	0.84	94.7
40–50	Peak height, Peak position, Coercivity, Integral area, Spectrum area	0.82	96.6

## Data Availability

Data sharing is not applicable to this article.
